# Genomic comparison of clinical strains of Mycobacterium shinjukuense in Japan reveals low diversity and stable genome structures

**DOI:** 10.1099/mgen.0.001695

**Published:** 2026-05-05

**Authors:** Takayuki Wada, Hiharu Inoue, Shiomi Yoshida, Yoshiro Murase, Yuriko Igarashi, Yukari Fukushima, Chie Nakajima, Yasuhiko Suzuki, Satoshi Mitarai

**Affiliations:** 1 Department of Microbiology, Graduate School of Human Life and Ecology, Osaka Metropolitan University, Osaka, Japan; 2 Osaka International Research Center for Infectious Diseases, Osaka Metropolitan University, Osaka, Japan; 3 Clinical Research Center, NHO Kinki Chuo Chest Medical Center, Sakai, Osaka, Japan; 4 Department of Mycobacterium Reference and Research, Research Institute of Tuberculosis, Japan Anti-Tuberculosis Association, Kiyose, Tokyo, Japan; 5 Division of Bioresources, International Institute for Zoonosis Control, Hokkaido University, Sapporo, Japan; 6 Division of Research Support, Institute for Vaccine Research and Development, Hokkaido University, Sapporo, Japan; 7 Department of Basic Mycobacteriology, Graduate School of Biomedical Science, Nagasaki University, Nagasaki, Japan

**Keywords:** genome comparison, genome epidemiology, genome stability, *Mycobacterium shinjukuense*, *Mycobacterium tuberculosis*-associated phylotype (MTBAP)

## Abstract

*Mycobacterium shinjukuense*, a rare non-tuberculous mycobacterial species closely related to *Mycobacterium tuberculosis*, remains poorly characterized at the genomic level. To obtain insights into its intraspecies genomic diversity and structure variation, we performed whole-genome sequencing on 18 clinical strains of *M. shinjukuense* collected from Japan between 2010 and 2017. Phylogenetic analysis revealed limited overall genetic diversity, with several clonal clusters likely representing identical strains. The phylogeny exhibited a largely star-like topology with few single nucleotide variations accumulated over time, suggesting a clonal expansion. Complete genome sequencing of five representative strains revealed high synteny, with no large-scale rearrangements, suggesting stable genomic structures. One strain, MSJ-01, harboured a unique 30 kb insertion region, possibly acquired via horizontal gene transfer. These findings provide insights into the genomic diversity and structure of *M. shinjukuense*, suggesting both its evolutionary stability and its potential to incorporate foreign DNA. This work expands upon available genomic resources and supports the utility of *M. shinjukuense* as a model for understanding genome evolution in the *M. tuberculosis*-associated phylotype lineage.

**Impact Statement**

This study provides the first comprehensive genomic characterization of *Mycobacterium shinjukuense*, a member of the *Mycobacterium tuberculosis*-associated phylotype (MTBAP). Comparative analyses of clinical strains isolated across different regions and years in Japan revealed remarkably low genetic diversity and conserved genome architecture, suggesting a stable lineage structure. Notably, one strain contained a unique insertion region likely acquired through horizontal gene transfer, indicating that even genetically stable MTBAP members may undergo occasional genetic acquisition. These findings advance our understanding of the evolutionary dynamics within the MTBAP group and highlight the importance of continued genomic surveillance of emerging mycobacteria.

## Data Summary

Five complete genome sequences of *Mycobacterium shinjukuense* were deposited at the International Nucleotide Sequence Database Collaboration using the auto-annotation tool, DDBJ Fast Annotation and Submission Tool (DFAST) [1], under the accession numbers AP038764–038768. Raw sequence data are available at the DNA Data Bank of Japan (DDBJ) Sequence Read Archive (DRA) under the accession numbers DRR597842–597859 (Illumina) and DRR597860–597864 (PacBio).

## Introduction

*Mycobacterium shinjukuense*, a member of the *Mycobacterium tuberculosis*-associated phylotype (MTBAP) [2], was first reported from Japan as a causative agent of non-tuberculous mycobacterial (NTM) infection [3]. Although *M. shinjukuense* is phylogenetically similar to *M. tuberculosis*, it remains clinically rare and undercharacterized. Moreover, *M. tuberculosis* is a well-studied global pathogen, whereas *M. shinjukuense* has been clinically reported only sporadically as a causative agent of NTM infections, mainly from Japan [4,8].

Because of its similarity to *M. tuberculosis*, *M. shinjukuense* serves as a potentially informative model for understanding the genomic traits relevant to *M. tuberculosis* evolution and pathogenicity. Recent phylogenomic analyses have placed *M. shinjukuense* near *Mycobacterium decipiens* within the MTBAP [9], suggesting its position on an evolutionarily intermediate branch. However, despite the potential significance of *M. shinjukuense*, their genomic data are limited. In particular, all existing genome-based analyses have relied solely on the type strain JCM 14233^T^ (also known as DSM 45633 and CCUG 53584), and no prior studies have examined the genetic diversity or population structure of clinical strains.

Cases of *M. shinjukuense* infection have been reported only from Japan and South Korea; *M. shinjukuense* causes chronic pulmonary disease with clinical features similar to those of tuberculosis [4,10]. In contrast to other MTBAP species, many of which are associated with extrapulmonary infections [9], *M. shinjukuense* appears to be restricted to pulmonary infections [3,9]. Notably, *M. shinjukuense* possesses a relatively compact genome (~4.5 Mb), comparable in size to that of *M. tuberculosis*, unlike the larger genomes observed in other MTBAP members (>5 Mb). Interestingly, these larger genomes in MTBAP species have been discussed as potential factors for environmental persistence or extrapulmonary dissemination [9]. Together, these features suggest that *M. shinjukuense* may have undergone genome reduction during adaptive evolution to the pulmonary niche, similar to *M. tuberculosis*. This perspective highlights the unique potential of *M. shinjukuense* to provide distinct insights into the evolution of pathogenicity within the MTBAP lineage, leading to *M. tuberculosis*, beyond what other MTBAP species offer.

Owing to its clinical rarity, the optimal treatment for *M. shinjukuense* infections remains uncertain. Common treatment regimens include a combination of clarithromycin, rifampicin and ethambutol, similar to those used for other NTM infections. Resistance to clarithromycin has been reported in certain cases, raising concerns regarding the potential need for alternative treatment strategies [4]. The diagnosis of *M. shinjukuense* is challenging because of its genetic similarity to *M. tuberculosis*, often requiring precise species-level identification via genetic sequencing [10].

In this study, we sought to address the gap in the genomic knowledge regarding *M. shinjukuense* by comprehensively sequencing the genomes of 18 clinical strains of *M. shinjukuense* isolated from patients across various regions of Japan over 8 years (from 2010 to 2017). In particular, our aim was to establish a broader view of intraspecies genomic diversity among clinical strains of this species, including mutation-level variation and large-scale genome structure, based on limited comparative genomic data.

## Methods

Clinical strains of *M. shinjukuense* used in this study were obtained through routine diagnostic and reference laboratory activities in Japan between 2010 and 2017, without collecting patients’ information. They were recovered from respiratory specimens, including sputum and bronchial aspirates, of patients with suspected or confirmed pulmonary mycobacterial infections and submitted for species identification and drug susceptibility testing. The collection reflects available cases during the study period and was not based on population-based sampling.

Genomic DNA of each strain was purified according to the method described by Murase *et al.* [11]. Genomic libraries were prepared using a QIAseq FX DNA Library Kit (Qiagen) following the manufacturer’s protocol. Whole-genome sequencing (WGS) was performed for all strains using the MiSeq system (Illumina) with MiSeq Sequencing Kit v3 (600 cyc); the protocol followed for WGS of *M. shinjukuense* was the same as that followed for *M. tuberculosis* [12]. Raw reads were processed using fastp (version 0.24.0) [13].

To analyse genetic relatedness among the *M. shinjukuense* strains, JCM 14233^T^ was used as a reference for mapping to detect single nucleotide variants (SNVs) and insertion/deletion (indels); this method allowed for a detailed examination of intraspecies genetic variation. SNV and indel filtering were performed using BactSNP (version 1.1.0) [14] under the pipeline platform SNPcaster (https://github.com/leech-rr/SNPcaster), according to Lee *et al.* [15], and CLC Genomics Workbench version 24.0.2 (Qiagen). The masked regions (Table S1, available in the online Supplementary Material) were determined by the ‘repeat-match’ command implemented in MUMmer (version 4.0.1) of the SNPcaster pipeline for detection of repeat regions and by longdust (version 1.4-r97: *https://github.com/lh3/longdust*) [16] for efficient detection of low-complexity regions. All SNVs and indels were checked and manually curated to remove mis-mapped positions due to non-specific mapping by visualizing the mapping context. Finally, they are listed in Table S2. A median-joining unrooted tree was constructed by using concatenated alleles of SNVs with PopART version 1.7 [17].

Long-read data were assembled using HGAP version 3.0 using the default settings. Each single contig was assembled from five strains by the assembly, followed by three rounds of polishing using Pilon version 1.22 [18] with short reads of each strain. Finally, the polished contigs were circularized with the long-read data respective to the strains by Circulator version 1.5.3 [19].

## Results and discussion

**Fig. 1.**
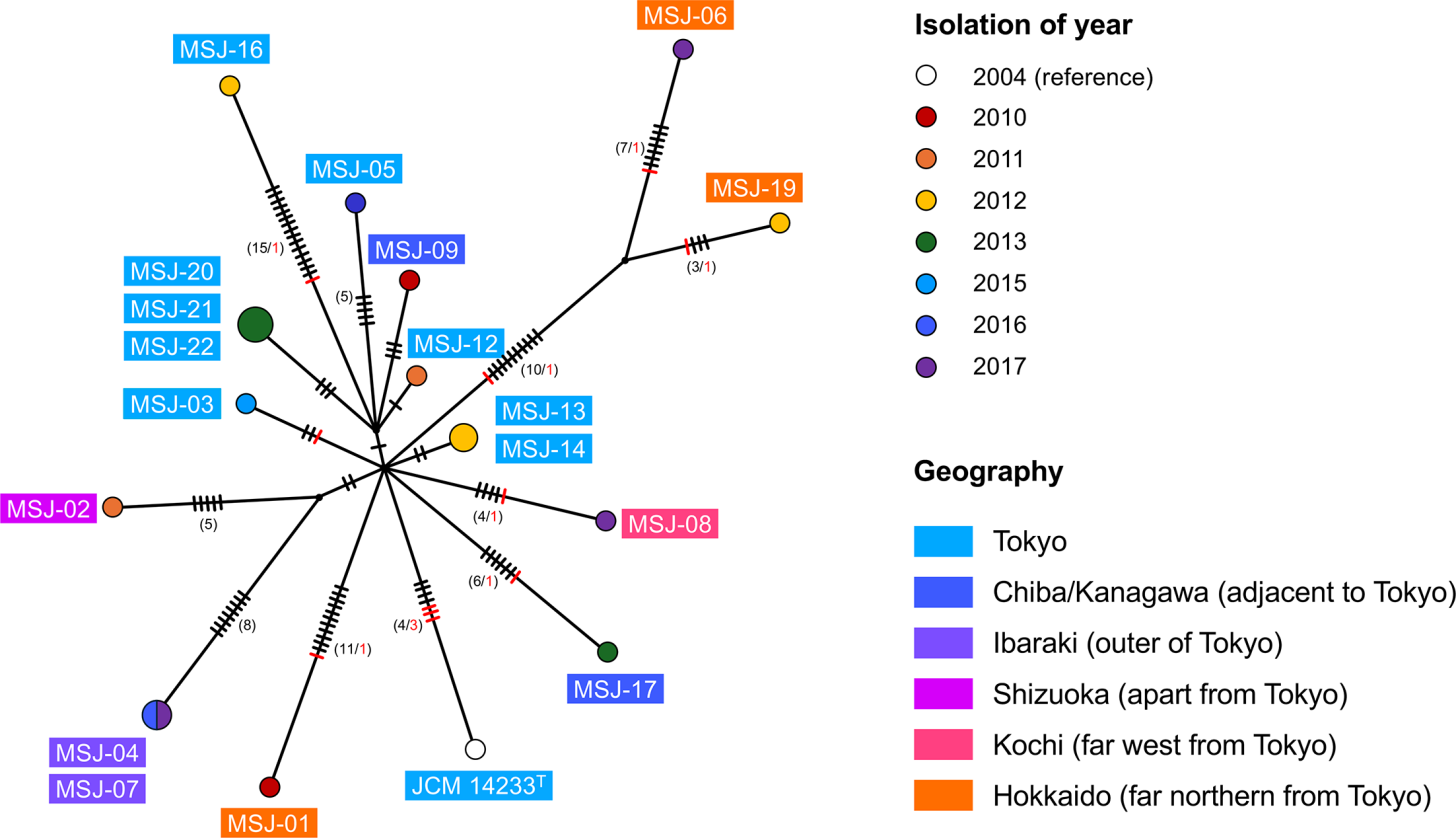
An unrooted median-joining tree was constructed based on the SNVs and indels identified using the 18 strains of *M. shinjukuense*. The size and colour of each node correspond to the number of strains and year of isolation, respectively. Strain names are shown in text boxes, with colours indicating their geographic origins. The hatches on each branch represent the number of SNVs (black) and indels (red) between the nodes. Numbers in parentheses also represent their numbers more than three, to make for greater clarity. Detailed information on the mutations is listed in Table S1.

Phylogenetic analysis revealed limited genetic diversity among the 18 *M*. *shinjukuense* strains (Fig. 1). Several clonal clusters were identified, including the strains such as MSJ-04 and MSJ-07 (both from Ibaraki Prefecture), MSJ-13 and MSJ-14 (both from Tokyo in 2012) and MSJ-20, MSJ-21 and MSJ-22 (all from Tokyo in 2017), all of which showed no SNVs within each cluster. These clusters shared geographic and temporal backgrounds, suggesting that the infections may be caused by a common bacterial clone. Across all 18 strains, pairwise SNV distances ranged from 0 to 33. Among the ten strains isolated from Tokyo, nine exhibited pairwise SNV distances of ≤10, supporting a highly clonal population in this region. However, low divergence was also observed among some strains from other prefectures, indicating that such clonality is not restricted to Tokyo but may reflect limited genomic diversity within *M. shinjukuense* across Japan. These findings highlight the overall genetic conservation of this species, despite its detection across multiple locations and years.

Topologically, the phylogenetic tree exhibited a star-like pattern, with multiple strains radiating from a central node without branching. This structure may reflect a past clonal expansion event, as discussed in genomic studies on *M. tuberculosis* [20], such as a localized outbreak or a bottleneck followed by dissemination. However, the actual timing of the most recent common ancestor remains uncertain, as only 2–17 SNPs were observed from the central node despite an 8-year sampling period, affecting robust inference of evolutionary timescales.

Despite this overall clonality, the detected variants (Table S2) revealed several non-synonymous mutations in genes associated with the ESX-1 secretion system, which has been discussed as a potential virulence determinant in MTBAP evolution [21]. Specifically, a frameshift mutation in *espH* and a non-synonymous substitution in *eccC* were observed in strain MSJ-01, while a point mutation in *eccCa1* was detected in strain MSJ-06. Although the functional impact of these mutations remains unknown, their presence indicates that virulence variation may occur even in genetically homogeneous populations in the species.

**Fig. 2.**
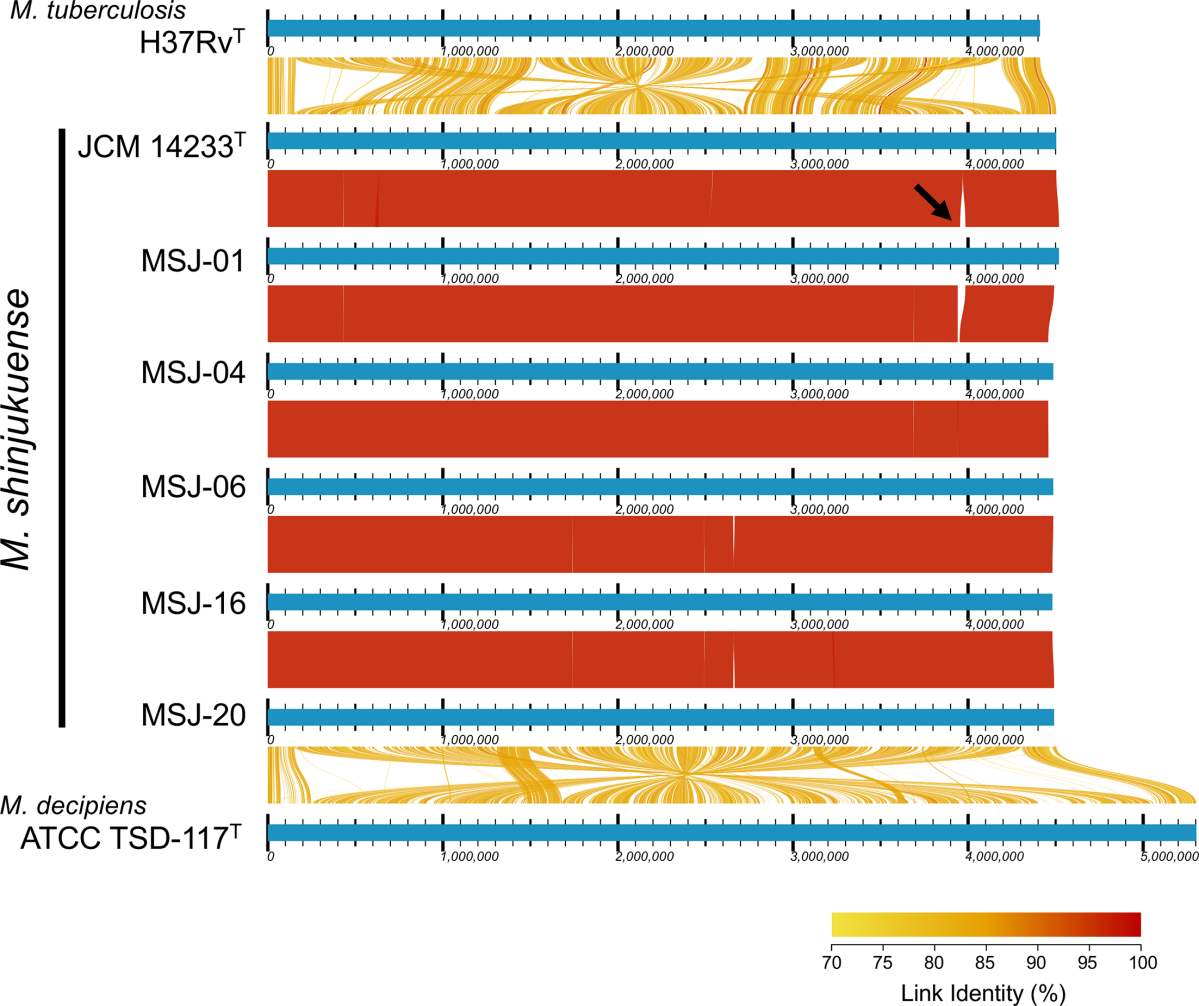
Genome alignments of six *M. shinjukuense* strains and *M. tuberculosis* H37Rv. Coloured ribbons (links) between genome tracks represent syntenic alignments, identified with LASTZ via the AliTV perl interface (v1.0.6) and visualized with the AliTV viewer (v1.0.6-74-g38a7398) [23]; link colour encodes pairwise nucleotide identity (see colour scale). Intraspecific ribbons are shown only for alignments wider than ~50,000 bp to reduce artificial signals, whereas interspecific ribbons are shown without length restriction to illustrate overall homology between species. Tracks are ordered relative to the *M. shinjukuense* ATCC 14233^T^ reference, and rearrangements are shown across the *M. tuberculosis* H37Rv and *M. decipiens* ATCC TSD-117^T^. The black arrow indicates a unique insertion region found only in strain MSJ-01.

Although the limited diversity among the *M. shinjukuense* strains suggests a conserved genetic background, whether this homogeneity extends to a large-scale genomic structure has not been previously examined. To explore the genomic construction of *M. shinjukuense*, we selected five representative strains for complete genome sequencing using the PacBio platform, which provides high-fidelity long reads. Complete genome assemblies revealed highly conserved synteny among the strains, with no large-scale rearrangements (Fig. 2). None of the strains harboured any plasmid. This structural conservation among *M. shinjukuense* strains parallels that observed in *M. tuberculosis* [22], suggesting that genomic stability may be a shared feature within each MTBAP species. The genomic stability of other MTBAP species, including *M. decipiens*, *Mycobacterium lacus* and *Mycobacterium riyadhense*, remains unexplored because of their clinical rarity. Future comparative genomic studies could offer valuable insights into the evolutionary processes that lead to genomic stability in this group.

In Fig. 2, the genomic distances between *M. shinjukuense* and two other MTBAP species, *M. tuberculosis* and *M. decipiens*, are also visualized. While *M. shinjukuense* and *M. tuberculosis* share a compact genome size (~4.5 Mb), *M. decipiens* retains a larger genome (>5 Mb), similar to other MTBAP species. Both species exhibit ~70% genome-wide identity to *M. shinjukuense*, indicating comparable levels of divergence among them. The fact that these three species are similarly distant from one another may provide a useful comparative framework for understanding genome evolution within the MTBAP lineage, regardless of differences in genome size. Notably, Sous *et al.* [21] indicated that *M. decipiens* exhibits suboptimal growth at 37 °C, favouring slightly lower temperatures. This physiological constraint may partly explain its limited association with pulmonary infections, in contrast to *M. tuberculosis* and *M. shinjukuense*.

**Fig. 3.**
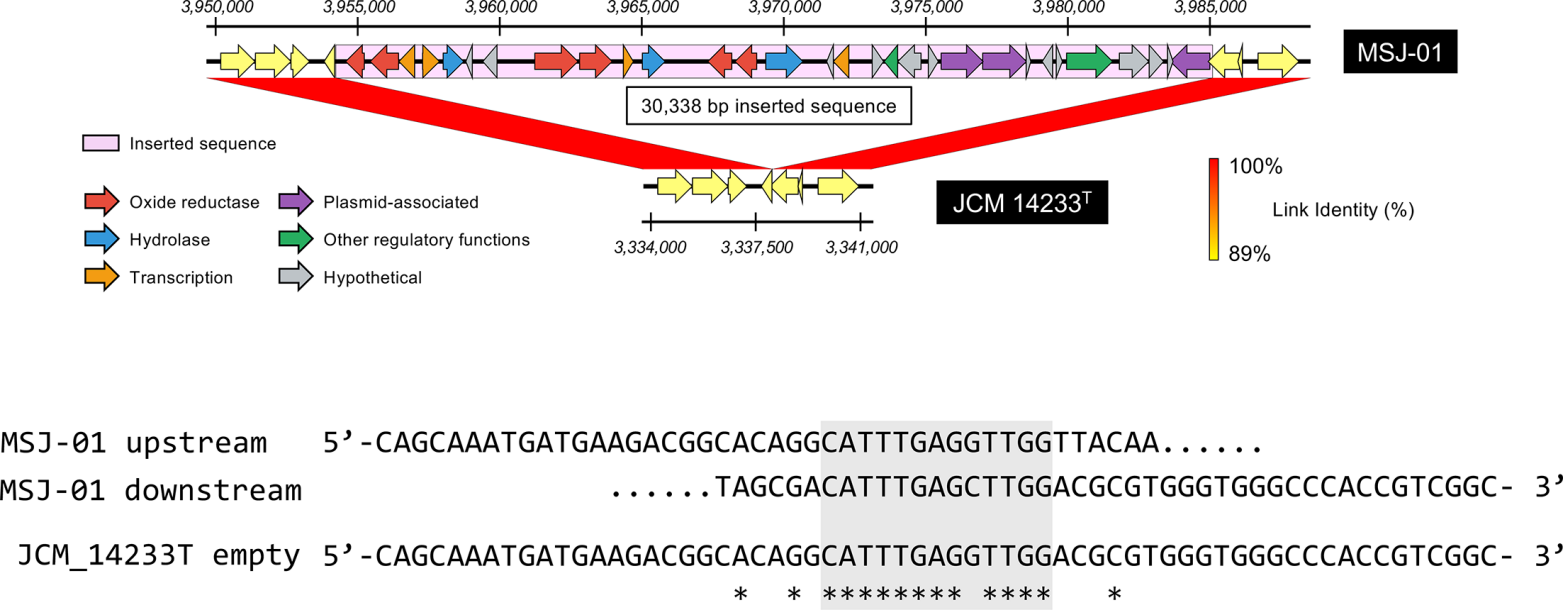
Schematic representation and sequence alignment of an insertion region in strain MSJ-01. (Upper) Synteny between the inserted region found in MSJ-01 and the corresponding locus in the reference strain *M. shinjukuense* ATCC 14233^T^. The insertion sequence spans 30,338 bp and contains 30 predicted coding sequences, including genes related to plasmid maintenance as well as those with oxidoreductase and hydrolase activities. This diagram was generated using Easyfig (version 2.2.5) [24]. (Lower) Nucleotide alignment of the flanking regions at the insertion site between MSJ-01 and ATCC 14233^T^. The 5′ and 3′ upstream sequences in MSJ-01 were aligned with the corresponding regions in the reference genome. A 14-bp overlapping sequence at the junction site is highlighted in a grey box.

Interestingly, one of the sequenced strains, MSJ-01, contained a unique insertion sequence of 30,338 bp not observed in any of the other strains (Fig. 2). Further analysis revealed a unique insertion region comprising ~30 genes (Fig. 3 and Table S3), encoding recombinases and DNA mobility-related proteins. blast analysis indicated that this insertion sequence has been found in other mycobacterial species, such as *Mycobacterium avium*, *Mycobacterium kansasii* and *Mycobacterium abscessus* (Table S4), suggesting acquisition of this genomic region via horizontal gene transfer (HGT) from other mycobacteria. A recent study has proposed that while the evolution of the *M. tuberculosis* complex has been primarily shaped by gene loss and point mutations, MTBAP species may have acquired genetic material via occasional HGT events [9], potentially contributing to early steps in human pathogenic adaptation. The insertion has not been previously characterized, and its origin remains uncertain. Although the functional significance and precise origin of the MSJ-01 insertion remain unclear, the presence of this horizontally acquired region suggests that *M. shinjukuense* may be permissive to the incorporation of foreign DNA.

Overall, our findings shed light on the genomic diversity and structural characteristics of *M. shinjukuense*. Phylogenetic analysis indicated that clinical isolates of *M. shinjukuense* collected from Japan exhibit low genetic diversity. Although the limited variation indicates a conservation of genomic structure, the large-scale structural features of this species are yet to be fully assessed. Complete genome sequencing confirmed a high level of synteny, reinforcing the viewpoint of structural stability across strains. Notably, one strain, MSJ-01, harboured a unique insertion region, likely acquired via HGT. Although the functional effect of this region remains unclear, its presence demonstrates that gene acquisition events can occur even within a genetically homogeneous population. Further studies are required to evaluate whether these elements contribute to ecological adaptation or have pathogenic potential.

## Supplementary material

10.1099/mgen.0.001695 Uncited Fig. S1.

10.1099/mgen.0.001695 Uncited Table S1.

10.1099/mgen.0.001695 Uncited Table S2.

10.1099/mgen.0.001695 Uncited Table S3.

10.1099/mgen.0.001695 Uncited Table S4.

## Abbreviations

HGT: horizontal gene transfer
MTBAP: *Mycobacterium tuberculosis*-associated phylotype
NTM: non-tuberculous mycobacterial
SNVs: single nucleotide variants
WGS: whole-genome sequencing
