# Supplementary figures and images for: Genomic comparison of clinical strains of Mycobacterium shinjukuense in Japan reveals low diversity and stable genome structures
Source: Microb Genom. 2026 May 5;12(5):001695. doi: 10.1099/mgen.0.001695 (PMC13143225; doi:10.1099/mgen.0.001695)

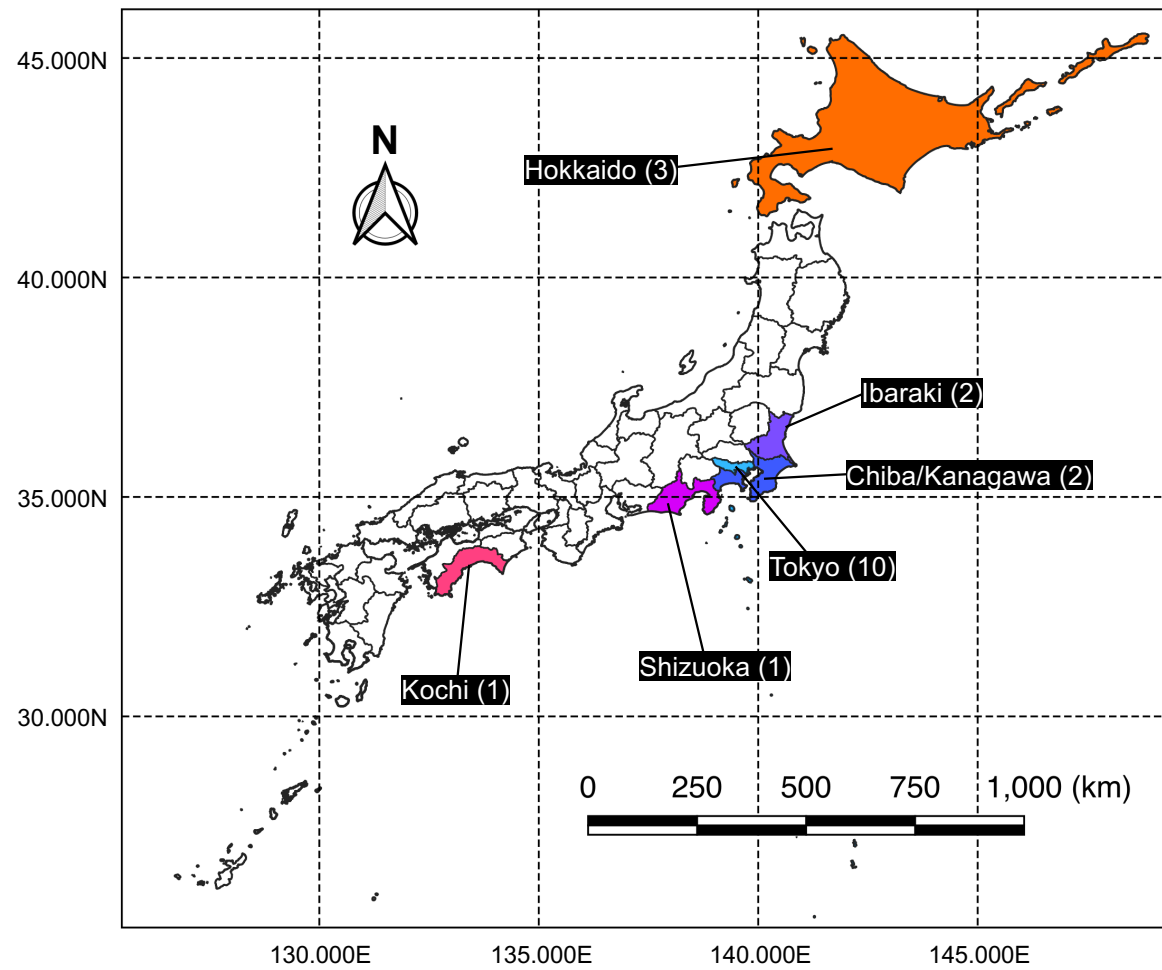

Wada et al., Fig. S1

Supplement: Uncited Fig. S1. [file mgen-12-01695-s001.pdf]
